# Theta Oscillations Are Sensitive to Both Early and Late Conflict Processing Stages: Effects of Alcohol Intoxication

**DOI:** 10.1371/journal.pone.0043957

**Published:** 2012-08-27

**Authors:** Sanja Kovacevic, Sheeva Azma, Andrei Irimia, Jason Sherfey, Eric Halgren, Ksenija Marinkovic

**Affiliations:** 1 Department of Radiology, University of California San Diego, La Jolla, California, United States of America; 2 Interdisciplinary Program in Neuroscience, Georgetown University, Washington, DC, United States of America; 3 Department of Neurology, University of California Los Angeles, Los Angeles, California, United States of America; 4 Graduate Program for Neuroscience, Boston University, Boston, Massachusetts, United States of America; California Pacific Medicial Center Research Institute, United States of America

## Abstract

Prior neuroimaging evidence indicates that decision conflict activates medial and lateral prefrontal and parietal cortices. Theoretical accounts of cognitive control highlight anterior cingulate cortex (ACC) as a central node in this network. However, a better understanding of the relative primacy and functional contributions of these areas to decision conflict requires insight into the neural dynamics of successive processing stages including conflict detection, response selection and execution. Moderate alcohol intoxication impairs cognitive control as it interferes with the ability to inhibit dominant, prepotent responses when they are no longer correct. To examine the effects of moderate intoxication on successive processing stages during cognitive control, spatio-temporal changes in total event-related theta power were measured during Stroop-induced conflict. Healthy social drinkers served as their own controls by participating in both alcohol (0.6 g/kg ethanol for men, 0.55 g/kg women) and placebo conditions in a counterbalanced design. Anatomically-constrained magnetoencephalography (aMEG) approach was applied to complex power spectra for theta (4–7 Hz) frequencies. The principal generator of event-related theta power to conflict was estimated to ACC, with contributions from fronto-parietal areas. The ACC was uniquely sensitive to conflict during both early conflict detection, and later response selection and execution stages. Alcohol attenuated theta power to conflict across successive processing stages, suggesting that alcohol-induced deficits in cognitive control may result from theta suppression in the executive network. Slower RTs were associated with attenuated theta power estimated to ACC, indicating that alcohol impairs motor preparation and execution subserved by the ACC. In addition to their relevance for the currently prevailing accounts of cognitive control, our results suggest that alcohol-induced impairment of top-down strategic processing underlies poor self-control and inability to refrain from drinking.

## Introduction

Oscillatory activity in theta (∼4–7 Hz) range in human electroencephalographic (EEG) recordings during cognitive tasks is maximal over fronto-midline scalp regions and has been termed fm-theta [1], [2]. Increased fm-theta power is associated with engagement of executive functions during cognitive inhibition [3], [4], [5] and in response to higher working memory load [6], [7], and errors [8]. Human intracranial studies indicate that fm-theta oscillations are generated in the anterior cingulate cortex (ACC) [9], [10], [11], confirming source modeling of scalp EEG sources to ACC or surrounding medial prefrontal cortex [12], [13], [14], [15]. However, because of their low spatial resolution, EEG estimates may be insensitive to other local theta generators such as the frontal and parietal cortices, revealed by intracranial recordings [11], [16], [17]. Indeed, extensive fMRI-based evidence indicates that various executive tasks activate an overlapping network including medial and lateral prefrontal and lateral parietal cortices [18], [19], [20], [21]. ACC, also termed rostral cingulate zone [22], seems to be the nexus of this distributed network and is particularly activated by conflict-inducing conditions that rely on controlled processing [23], [24], [25].

Alcoholics and children at risk for developing alcoholism manifest reduced event-related theta power during cognitive tasks [26], [27], [28]. Furthermore, both event-related theta power and alcohol dependence have been linked to the same alleles in genes coding for neurotransmitter receptors [29]. Given this converging evidence, the scarcity of studies exploring effects of acute intoxication on event-related theta power is notable. In a study of auditory memory, Krause and colleagues [30] showed that event-related fm-theta is attenuated by a moderate alcohol dose.

A recent fMRI study [31] showed that moderate intoxication selectively attenuates ACC activation during high-conflict and error trials during the Stroop naming task, indicating vulnerability of regulatory, top-down functions to alcohol. However, the spatio-temporal characteristics of this effect remain unexplored. More specifically, it is not clear which processing stage is most affected by alcohol and what are the relative contributions of the ACC and the fronto-parietal distributed network under alcohol intoxication. It is possible that the ACC is primarily active during the early processing stage as it monitors for potential conflict [23], [24]. It may also contribute to response inhibition, selection, and execution [31]. Data analysis methods adapted to the time-sensitive signal allowed us to examine alcohol’s effects on both, stimulus-related processing and response preparation stages. The aim of the present study was to use multimodal imaging to investigate the effects of a moderate alcohol dose on total event-related theta power during different stages of conflict processing elicited by the Stroop naming task in healthy participants. Better insight into the dynamics of relative contributions to successive processing stages is essential for the currently prevailing accounts of the specific roles that the ACC and fronto-parietal areas play during decision conflict. Furthermore, better understanding of alcohol’s effects on regulatory functions may illuminate alcohol-induced dysregulation of self-control and inability to desist drinking, which can contribute to alcohol dependence [32], [33], [34].

## Results

### Behavioral Measures

#### Performance

**Figure 1:**
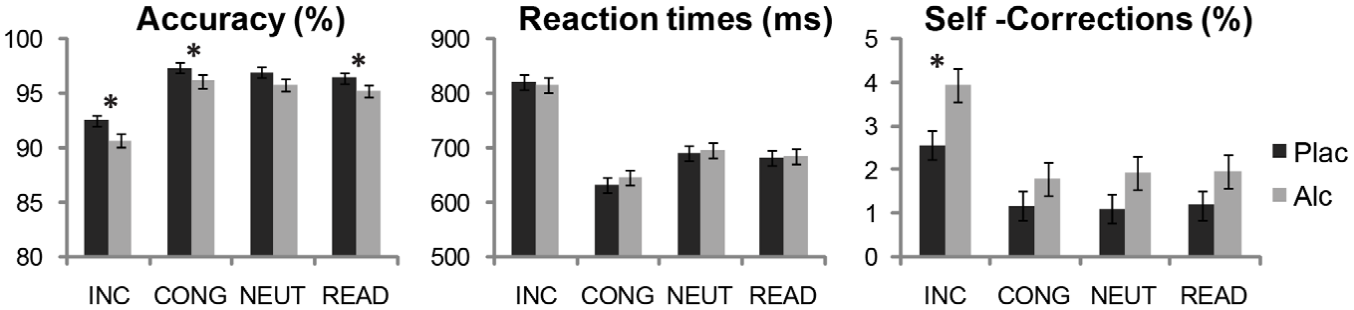
Performance measures. Accuracy, reaction times, and the percentage of trials on which participants corrected their responses immediately after an error (means ± standard errors) are shown for each task and beverage condition. The Stroop interference effect is indicated by lower accuracy and slower reaction times to conflict-inducing incongruous (INC) stimuli overall. Alcohol did not affect RTs, but participants responded less accurately and made more corrective responses on INC trials when intoxicated. Significant alcohol vs. placebo comparisons for each condition are marked, *p<0.05.

The modified Stroop task successfully evoked interference effect as indicated by lower accuracy (F(1,19) = 40.8, p<0.0001 ) and longer reaction times (F(1,19) = 130.5, p<0.0001) to INCONG as compared to all other conditions, Fig. 1. Subjects were more accurate under placebo than alcohol as indicated by the main effect of beverage (F(1,19) = 12.3, p<0.005). There was no main effect of beverage or beverage by condition interaction on RTs. However, there was a strong main effect of condition (F(3,57) = 82.5, p<0.0001), with the slowest responses to INCONG (820±126 ms). Responses to CONG were the fastest (639±89 ms), differing from both RTs to NEUT (693±98 ms, F(1,19) = 81.7, p<0.0001) and READ (683±100 ms, F(1,19) = 12.9, p<0.005). Participants made more corrective responses on INCONG trials compared to all other conditions (χ^2^ = 4.8, p<0.05), and more under intoxication compared to placebo especially on INCONG trials (χ^2^ = 4.5, p<0.05), Fig. 1. Reduced accuracy under alcohol correlated with three measures of impulsivity: psychoticism scale of Eysenck’s Personality Questionnaire (r = −0.59, p<0.001), impulsivity scale on Eysenck’s Impulsiveness and Venturesomeness Scale (r = −0.54, p<0.02), and thrill and adventure seeking scale of Zuckerman Sensation Seeking Scale (r = −0.58, p<0.02), suggesting that alcohol may impair the ability to inhibit the prepotent but erroneous responses.

#### Post-experimental questionnaire and mood ratings

At the end of each experimental session participants were asked to provide ratings of various aspects of their subjective experience using Likert scales. On the scale from 1 (definitely contains no alcohol) to 5 (definitely contains alcohol) subjects rated the beverage contents as 4.7±0.5 under alcohol and significantly lower under placebo, 1.3±0.4, χ2 = 20.0, p<0.0001. Participants estimated that the alcoholic beverage contained 2.3±0.7 “alcoholic drinks” which was a slight underestimate of the actual average amount containing 2.8 standard drinks defined as 1.5 fl oz of vodka. In contrast, they estimated that the placebo beverage contained 0.0±0.1 “alcoholic drinks”. On the scale from 1 (not at all) to 5 (very much), participants reported feeling moderately intoxicated (2.7±0.7) under alcohol, but not at all intoxicated under placebo (1.0±0), χ^2^ = 20.0, p<0.0001. They were only slightly more dizzy under alcohol than placebo (1.8±0.8 vs. 1.1±0.2), but this difference was significant, χ2 = 12.0, p<0.001. On the scale from 1 (easy) to 5 (difficult), subjects rated the task as being moderately difficult (2.8±0.9) but the perceived difficulty was not influenced by beverage.

Mood ratings were available for 19 participants. Overall, they felt less stimulated and more sedated at the end of the recording, as indicated by the main effects of BrAC phase on the BAES stimulation (F(2,36) = 6.1, p<0.005) and sedation subscales (F(2, 36) = 3.6, p<0.05). Baseline ratings that were obtained prior to beverage administration did not differ between sessions thus they were subtracted from the ratings obtained at the ascending and descending BrAC limbs. Relative to baseline, subjects reported feeling more stimulated on the ascending phase of BrAC under alcohol than under placebo (F(1,18) = 9.3, p<0.01). There were no main effects of interactions for the sedation BAES scale.

### MEG Results

**Figure 2:**
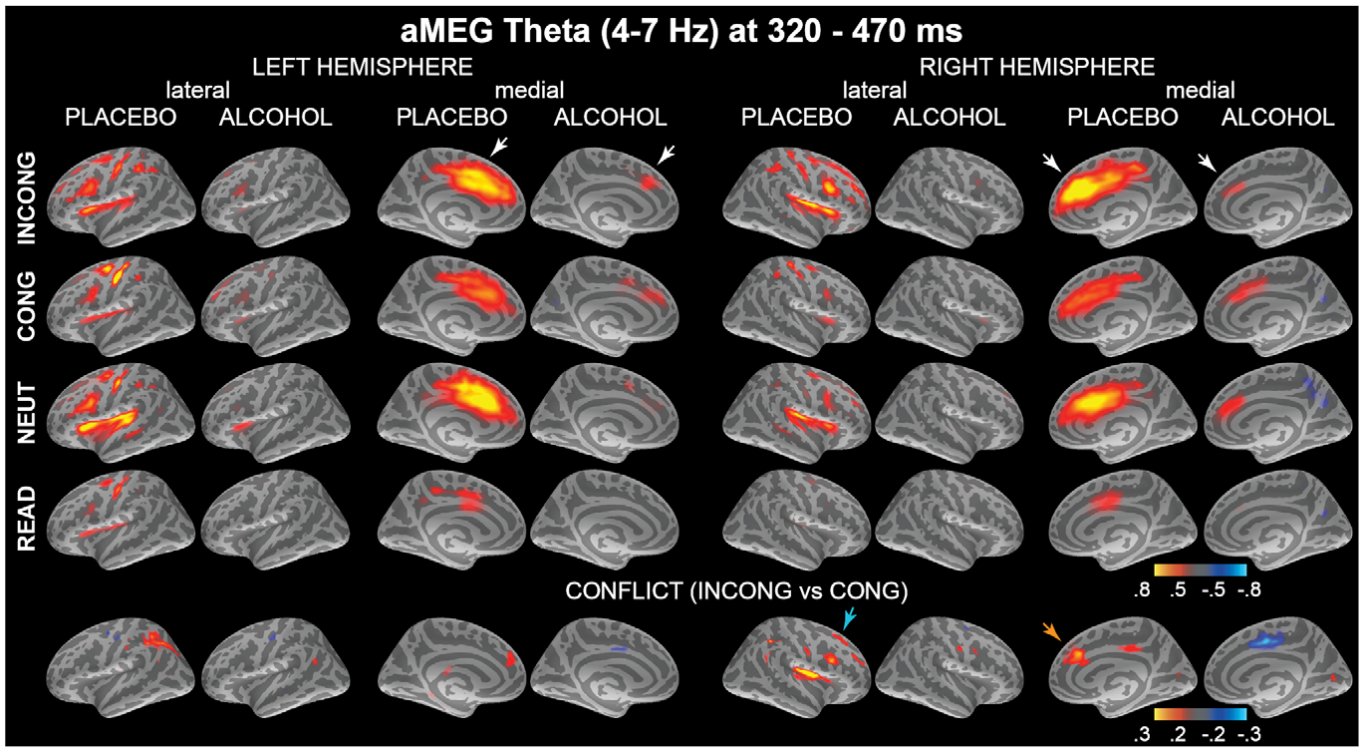
Group-average maps of event-related theta source power estimates in 320–470 ms time window. Event-related theta power is elicited in the fronto-parieto-cingulate network with the ACC as the strongest estimated source, and is attenuated by intoxication (*white arrows*). The color scale depicts baseline-corrected noise-normalized source power expressed in arbitrary units. The bottom row shows conflict–related theta power (INCONG - CONG contrast) for both beverage conditions. The color scale denotes differential baseline-corrected source power estimates, with red-yellow indicating stronger theta power to INCONG. Conflict-related theta is attenuated by intoxication in the right prefrontal (*cyan arrow*) and ACC (*orange arrow*) cortices. CONG stimuli elicited stronger theta in the motor -related medial cortex due to motor preparation at this latency.

**Figure 3:**
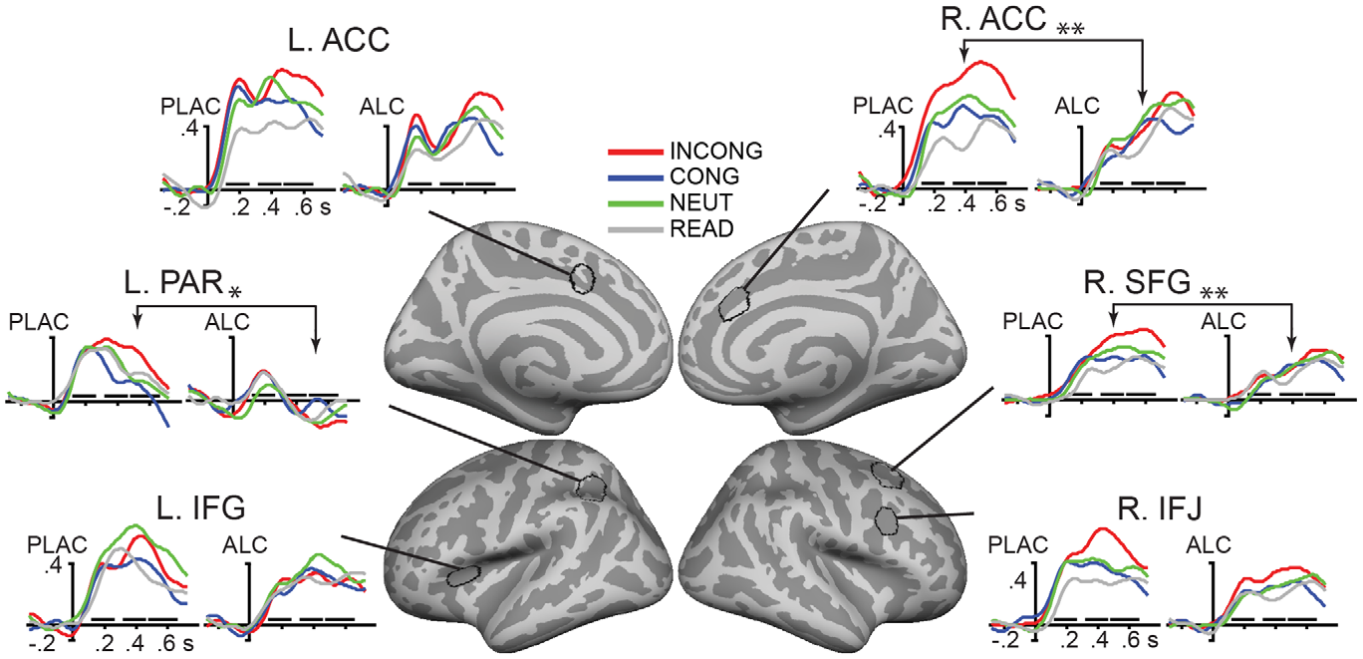
Group-average timecourses of event-related theta source power estimates in selected regions of interest. While alcohol reduces event-related theta power overall, attenuation of the conflict-related theta (INCONG vs. CONG contrast) is particularly prominent in ACC, with contributions from lateral fronto-parietal areas. Direct comparison of the beverage effects on conflict-related theta reached significance as indicated by arrows, *p<0.05, **p<0.01. Horizontal bars indicate the three time windows for which power was averaged and entered into statistical analysis. The y-axis depicts baseline-corrected noise-normalized source power expressed in arbitrary units. ACC: anterior cingulate cortex; IFJ: inferior frontal junction; IFG: inferior frontal gyrus; SFG: superior frontal gyrus; PAR: parietal cortex.

The Stroop task increased total event-related theta power in a distributed fronto-parietal cortical network as shown in Fig. 2, with the ACC being the strongest estimated generator of theta activity. Alcohol inebriation attenuated event-related theta power overall. Conflict-related increase in theta power (i.e. INCONG vs. CONG contrast) was observed only under placebo but not under alcohol in the cognitive control network including ACC, lateral frontal, and parietal regions. Timecourses of averaged event-related theta power estimates in the ROIs representing the main foci of theta power are presented in Fig. 3 and the results of ANOVAs in Table 1.

**Table 1:** Summary of ANOVAs of event-related theta for different ROIs (MEG), and Fz and Cz electrode locations (EEG).

				Conflict: INCONG - CONG		
	Condition	Beverage	Condition X Beverage	ALC	PLAC	ALC-PLAC
	F(3,57)	F(1,19)	F(3,57)	F(1,19)	F(1,19)	F(1,19)
**MEG Results**						
**T2 (320–470 ms)**						
L. ACC	4.4**	8.5**	0.9	0.3	1	1
L. IFG	4.2**	5.2*	1.8	1.5	9.1**	2.3
L. PAR	1.8	12.4**	1.6	0.1	8.3**	3.8
R. ACC	9.8****	3.5†	4.9**	0.2	11.9**	9.9**
R. IFJ	8.8****	7.1*	1	2	10.5**	1.5
R. SFG	6.4***	6.0*	3.0*	0.1	13.4**	11.3**
**T3 (480–670 ms)**						
L. ACC	3.8*	0.8	0.4	2.7	5.1*	0
L. IFG	5.5**	0.6	2.8*	2	13.8**	2.5
L. PAR	0.3	9.0**	2.9*	1.3	4.2†	5.7*
R. ACC	9.8****	0.2	2.3†	4.8*	34.1****	1.7
R. IFJ	4.4**	1.6	0.6	1.2	11.0**	0.8
R. SFG	5.8**	1.2	1.6	2.1	16.2***	3.1
**EEG Results**						
**T2 (320–470 ms)**						
Fz	2.6†	5.8*	0.3	0.4	0.1	0
Cz	1.2	12.4**	0.6	0	0.8	0.5
**T3 (480–670 ms)**						
Fz	5.3**	1.4	0.4	4.5†	8.3*	0
Cz	3.4*	3.3†	1	2.3	21.7***	1.4

Included are the results for main effects and interactions of Condition and Beverage, conflict-related theta power (INCONG - CONG contrast) for alcohol and placebo, and beverage effect on conflict-related theta power (Alc - Plac x INCONG - CONG interaction).

ACC: anterior cingulate cortex; IFJ: inferior frontal junction; IFG: inferior frontal gyrus; SFG: superior frontal gyrus; PAR: parietal cortex. Conflict-related theta power is larger to INCONG than CONG in all cases. Significance level is indicated as follows:

* p<0.05,

** p<0.01,

*** p<0.001,

**** p<0.0001;

† p<0.1.

#### T1 (120–270 ms)

The main effect of beverage was observed in most regions during the early time window, with stronger event-related theta power under placebo than alcohol overall. The only conflict-related effect was increased theta power in the right ACC under placebo (F(1,19) = 8.6, p<0.01) but not under alcohol (F(1,19) = 1.5, *ns*).

#### T2 (320–470 ms)

Conflict-related increase in theta power was observed under placebo but not alcohol in the right ACC as well as lateral frontal and parietal regions (Table 1, Fig. 2). Direct comparison of the beverage effects on the conflict-related theta power (Alc - Plac x INCONG - CONG interaction) showed significant attenuation by alcohol in the right ACC (F(1,19) = 9.9, p<0.01) and right SFG (F(1,19) = 11.3, p<0.005).

#### T3 (480–670 ms)

Bilateral ACC and right lateral frontal regions showed conflict-related increase in theta power under placebo, but not under alcohol (Table 1). The effect of beverage on conflict-related theta power was significant in the late time window in the left parietal region (F(1,19) = 5.7, p<0.05). Under alcohol, event-related theta power in the right ACC was negatively correlated with RTs to INCONG – the longer the RTs, the weaker theta (r = −0.65, p<0.005).

Not all frontal regions showed sensitivity to conflict. In the middle time window, left IFG showed the largest response to neutral stimuli as compared to all others under both placebo (F(1,19) = 8.0, p<0.02), and alcohol (F(1,19) = 10.2, p<0.005), in agreement with its role in semantic processing [35]. A similar effect was found in the late time window under placebo (F(1,19) = 12.5, p<0.005), but not under alcohol (F(1,19) = 2.2, *ns*).

#### Baseline theta

Total theta power in the prestimulus period was analyzed in order to examine the possibility that the observed effects of alcohol on event-related theta power are due to baseline changes. While there were no beverage or condition effects in any of the left hemisphere regions, the baseline theta was marginally reduced in the right ACC (F(1,19) = 3.7, p<0.1), IFJ (F(1,19) = 4.0, p<0.1), and SFG (F(1,19) = 4.6, p<0.05) under alcohol. Greater sensitivity of the right hemisphere to the effects of alcohol has been reported previously [36], [37], [38]. However, since event-related theta power is calculated by subtracting the baseline power from the raw power, a decrease in the baseline theta power under alcohol would result in increased event-related theta power. Therefore, it is unlikely that alcohol-induced reduction in event-related theta power is caused by changes in the baseline power, but it reflects task related changes in theta power independent from baseline effects.

#### Pre-response theta

**Figure 4:**
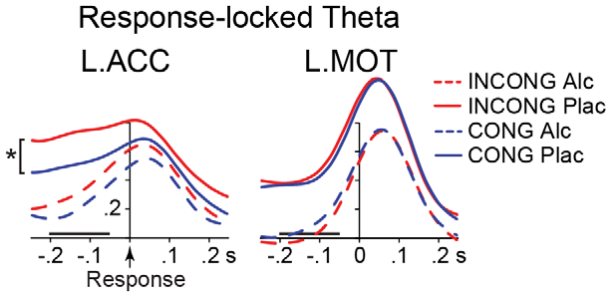
Group-average timecourses of response-locked theta source power estimates in the left ACC and MOT areas. Time 0 ms corresponds to the button press. The time window immediately preceding motor responses is indicated by a horizontal bar. During this time, only ACC showed sensitivity to conflict with stronger theta on INCONG than CONG trials during placebo (* p<0.02), suggesting the ACC engagement in response selection and execution. Alcohol attenuated pre-response theta overall. The y-axis depicts baseline-corrected noise-normalized source power expressed in arbitrary units.

Pre-response theta power was calculated for the −200 to −50 ms time window immediately preceding the motor response, time-locked to button presses (Fig. 4). It showed sensitivity to conflict in the ACC bilaterally only under placebo (left: F(1,19) = 6.6, p<0.02 right: F(1,19) = 6.7, p<0.02), but not alcohol (F(1,19) <0.6, *ns*). Alcohol reduced the response preparation theta power bilaterally in the ACC (left: F(1,19) = 8.8, p<0.01; right: F(1,19) = 4.4, p<0.05) and the motor cortex (left: F(1,19) = 8.0, p<0.02; right: F(1,19) = 7.0, p<0.02), indicating alcohol-induced impairment of the regions essential for motor preparation and execution. The effect of Beverage X Condition interaction was not significant in any of the ROIs. Theta to INCONG was negatively associated with RTs, r = −0.53, p<0.02, so that lower theta was associated with longer RTs to high-conflict stimuli.

### EEG Results

**Figure 5:**
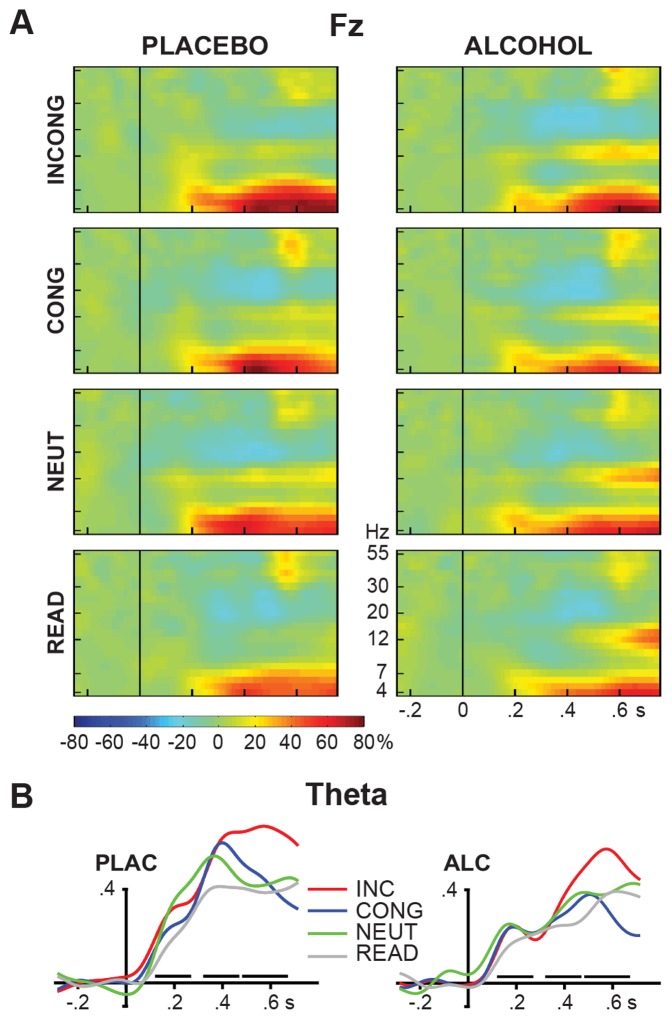
Grand averages of event-related EEG power at the frontal (Fz) electrode. a) Time-frequency plots of total event-related power expressed as the relative percentage change from the power in the baseline (−250 to 0 ms) for each frequency. Four task conditions (rows) are shown for placebo and alcohol sessions. Most pronounced beverage and task effects were observed in the theta band. b) Group averaged total event-related power in the theta frequency band at the Fz electrode. Horizontal bars indicate the three time windows for which power was averaged and entered into statistical analysis. Alcohol decreased total event-related theta power overall. Conflict-related increase in theta power was significant in the late time window only under placebo.

Grand average time-frequency plots of total event-related power, expressed as a change relative to the baseline period, at 4 to 55 Hz for the Fz electrode location are presented in Fig. 5a, and total event-related theta power timecourses in Fig. 5b. Repeated measures ANOVAs of the event-related theta power were carried out for the three latency windows as described above. While no effects were observed for the first time window (T1), alcohol reduced event-related theta power overall in the middle time window (T2), Table 1. INCONG stimuli elicited the largest event-related theta power in the late time window (T3) especially under placebo (Table 1). The conflict-related increase in theta power (i.e. INCONG vs CONG contrast) in T3 was significant under placebo (Fz: F(1,16) = 8.3, p<0.05; Cz: F(1,16) = 21.7, p<0.0005) but only marginally under alcohol (Fz: F(1,16) = 4.5, p<0.1; Cz: F(1,16) = 2.3, ns). Uncorrected theta power in the baseline period was unaffected by either beverage or condition.

## Discussion

This study has employed a time-sensitive multimodal imaging approach to examine the effects of alcohol intoxication on spatio-temporal characteristics of event-related total theta power as a function of the Stroop interference. The main findings can be summarized as follows: 1) The Stroop task elicits event-related theta power in a fronto-parietal network with the primary generator estimated to ACC. 2) Event-related theta power is stronger during conflict trials (i.e. INCONG vs. CONG contrast) under placebo. 3) During the early time window, only the ACC is sensitive to decision conflict, confirming its role in conflict detection. During subsequent processing stages the ACC remains the principal generator of the conflict-related theta power. While alcohol attenuates event-related theta power overall, conflict-related theta is reduced selectively by alcohol in ACC and lateral fronto-parietal regions. 4) In the interval immediately preceding the motor response, the ACC is the only region showing differential theta to conflict, suggesting that it additionally contributes to response control. Alcohol decreases theta in ACC to high-conflict INCONG stimuli during motor preparation which correlates negatively with reaction times. Longer RTs are associated with decreased theta power under alcohol, indicating that alcohol impairs ACC’s role in response preparation and execution under conflict conditions. 5) Results were pooled across both genders since men and women did not differ on any of the measures, in agreement with our previous fMRI study with the same task [31]. Overall, these results indicate that event-related theta power is reduced by moderate alcohol intoxication in ACC and fronto-parietal regions during conflict processing. Selective vulnerability of the top-down executive functions to alcohol may result in reduced self-restraint and propensity of engaging in continued or heavy drinking.

The anatomically-constrained distributed MEG approach applied in the current study estimated the principal generator of event-related theta oscillations to ACC, with other fronto-parietal areas additionally contributing to the signal (Fig. 2). This finding is in agreement with human intracranial studies confirming that ACC is a major generator of theta evoked by cognitive tasks [9], [10], [11]. Source localization EEG/MEG studies also estimate theta power to the ACC and the surrounding medial prefrontal area in a variety of demanding tasks [12], [14], [15], [39], [40], inclusive of the Stroop task [13]. However, methods with refined spatial resolution indicate that a fronto-parietal network additionally contributes to theta generation during cognitive tasks, including human intracranial [11], [16], [17], [41] and combined EEG/fMRI studies [42], [43].

In the present study, high-conflict (INCONG) stimuli evoked stronger theta power in ACC and fronto-parietal areas than other conditions under placebo. Conflict-related theta increase (defined as INCONG vs. CONG comparison) was observed only in the right ACC in the early time window (120–270 ms), suggesting its sensitivity to the early conflict detection stage. Evidence from studies using event-related potential (ERP) approach is broadly consistent with the timing of this difference, as it shows that the N2 component is sensitive to conflict [24], [44]. However, reports of alcohol’s effects on N2 during a flanker task are inconsistent [44], [45]. Because N2 and theta power are both clearly important for the early conflict detection stage, further studies are needed to resolve ACC’s contributions to these measures in the context of beverage effects on conflict-inducing conditions.

The effect of conflict was most pronounced during subsequent processing stages in ACC, but was also observed in other, primarily prefrontal areas (Table 1, Fig. 3). EEG data were in the overall agreement with the aMEG results, with the strongest increase in conflict-related theta power in the later time window (T3, 480–670 ms). These findings are consistent with a large number of EEG studies suggesting that fm-theta increases in response to more difficult task requirements [6], [7], [12], [15], response inhibition [3], [4], [5] and errors [8], [46]. The aMEG approach made it possible to estimate conflict-related theta to the ACC as the principle generator, with additional contributions from distributed fronto-parietal areas.

In addition to conflict detection, the ACC is also involved in response selection as specifically examined by time-locking theta power estimates to button-press responses. Only ACC showed sensitivity to conflict under placebo, with stronger theta on INCONG than CONG trials in the time window immediately preceding response execution (Fig. 4). This finding clearly implicates the ACC engagement in motor planning and execution which is made possible by its dense motor projections [47] and is especially evident under more difficult conditions [48]. Furthermore, theta power estimated to ACC correlated negatively with RTs under intoxication during the late time window encompassing response preparation. Slower RTs were associated with attenuated theta power in the ACC, suggesting that alcohol impairs motor preparation and execution subserved by the ACC. Therefore, ACC’s selective sensitivity to conflict starting with the early processing stage and its role in motor preparation and execution on high-conflict trials, reveal its top-down regulative and executive role in goal-oriented actions under conflict [22], [48], [49]. Widespread anatomical connections of the ACC with lateral prefrontal cortex, motor and limbic areas, make the ACC suitable for its multifaceted role in self-regulation [50], [51]. These results support the Conflict Monitoring account of ACC function [23], [24], confirming its role in cognitive control processes including conflict detection and response selection.

In the present study, alcohol intoxication decreased event-related theta power overall, but its selective effects on conflict-related theta (INCONG vs. CONG contrast) were evident starting with the earliest time window (Fig. 3). At this latency, conflict-related theta power was observed only in ACC under placebo and was blunted by alcohol, suggesting impairment of the early conflict detection stage as subserved by the ACC. Alcohol abolished conflict-related theta power increase in subsequent time windows particularly in the right ACC with superior prefrontal contributions, consistent with at least some evidence suggesting sensitivity of the right hemisphere to alcohol [38]. This finding is also compatible with results of our previous fMRI study employing the same paradigm and the same level of alcohol intoxication [31]. In that study, alcohol selectively attenuated BOLD activation in ACC during high-conflict trials and erroneous responses. These two neuroimaging methods rely on fundamentally different sources of signal: MEG/EEG signals reflect postsynaptic currents measured as magnetic fields and electric potentials [52], [53]. In contrast, blood oxygen level-dependent (BOLD) signal depends on hemodynamic changes, reflecting neural activity only indirectly as a result of neurovascular coupling [54]. Due to its limited temporal resolution, the BOLD signal cannot resolve differential effects on successive processing stages. Furthermore, the BOLD is sensitive to alcohol’s vasoactive properties and may not accurately represent the magnitude of the neural changes resulting from intoxication [55], [56]. Therefore, temporally sensitive methods are needed to verify alcohol’s effects on the executive network. Nevertheless, both aMEG and BOLD-fMRI results concur in showing that alcohol intoxication attenuates conflict-induced activation in ACC. Overall, this convergence of results strongly indicates that regulative functions are particularly vulnerable to moderate alcohol intoxication. Indeed, a large number of neuroimaging studies suggest that ACC subserves top-down controlled processing [22], [24], [49] in the sense of guiding behavior that is not habitual or automatic [25]. The present aMEG results further refine and extend this overall conclusion by providing insight into the successive *stages* of processing with respect to stimulus presentation as well as response execution. While ACC is uniquely sensitive to conflict and it is the principal generator of theta during both early conflict detection, and late response preparation and execution stages, it is co-active with other fronto-parietal areas that also contribute to theta generation. Alcohol intoxication attenuates theta associated with conflict indicating that the controlled processing is susceptible to its effects during successive stages. Controlled processing, an aspect of executive functions, refers to the capacity to inhibit automatic responses in favor of relevant, previously unrehearsed responses [57], [58], [59]. Its main function is to ensure that our actions can be flexibly modified in agreement with intents and goals [60]. Results of the present study indicate that alcohol disrupts the top-down strategic processing of conflict, potentially resulting in increased susceptibility to immediate cues. Indeed, impaired self-regulation is considered important in the development of alcohol abuse. Loss of control may result from reduced ability to refrain from drinking [33], [34], [61], [62], [63].

In sum, results of this study confirm the essential contribution of the ACC to executive regulation and its vulnerability to moderate alcohol intoxication, in agreement with a previous fMRI study using the same paradigm [31]. However, superior temporal sensitivity of the aMEG method employed here further refined and extended these findings by providing insight into alcohol effects on conflict processing stages. The principal generator of conflict-related theta power was estimated to ACC, with contributions from distributed fronto-parietal areas. The ACC was uniquely sensitive to conflict during both early, conflict detection, and later stages reflecting response preparation and execution, suggesting a broader functional role of the ACC in decision conflict than proposed by major theoretical accounts [23], [24]. Alcohol attenuated event-related theta across successive processing stages. Selective vulnerability of the top-down, regulative capacity to alcohol intoxication may contribute to impaired self-control and inability to desist drinking [32], [34]. These findings further support the importance of theta as a biomarker of vulnerability to alcohol dependence [29].

## Materials and Methods

### Ethics Statement

The study was approved by the Human Research Committee of the institution where the study was performed (Massachusetts General Hospital and the Partners Healthcare Network) and was conducted according to the principles expressed in the Declaration of Helsinki. Written informed consent was obtained from all participants involved in the study.

### Subjects

Twenty young, healthy volunteers (12 men, 8 women, mean±SD age = 25.3±4.4 years) successfully completed all sessions of the experiment. All participants were right-handed non-smokers and reported no medical, alcohol- or drug-related problems and no family history of alcohol or drug abuse in their first or second degree relatives. None used any medication at the time of the study and none reported any previous head-injuries or had any MRI contraindications. Subjects reported drinking alcohol occasionally (2.0±1.2 times a week) and in low-to-moderate amounts (2.6±1.1 drinks per occasion) in social settings. No alcoholism-related symptoms were detected with the Short Michigan Alcoholism Screening Test, SMAST [64]. Subjects were reimbursed for their participation.

### Experimental Design

All subjects participated in an introductory session during which they provided detailed information on their medical history, family history of alcoholism, level of response to alcohol (Self-Rating of the Effects of Alcohol, SRE) [65], severity of their alcoholism-related symptoms (Short Michigan Alcoholism Screening Test, SMAST) [64], quantity and frequency of alcohol use [66], and handedness [67]. In order to obtain a comprehensive dispositional profile for each subject particularly with respect to disinhibitory, novelty seeking and socialization traits, the following questionnaires were used: Eysenck Personality Questionnaire, EPQ [68], Eysenck Impulsiveness and Venturesomeness Scale [69], Zuckerman Sensation Seeking Scale [70]. In addition, the purpose of the initial, non-experimental visit to the laboratory was to familiarize participants with the laboratory setting and the experimental procedure, abating potential effects of situation-induced arousal [71].

Subjects subsequently participated in both alcohol and placebo MEG sessions in a counterbalanced manner. The within-subject design minimized influence of individual differences in anatomy, alcohol metabolism, and brain activation patterns, resulting in reduced error variance and increased statistical power. Placebo and alcohol sessions were 32±26 days apart on average. Urine pregnancy test administered to women in the beginning of each session ascertained that none were pregnant. All subjects were asked about their compliance with requirement to abstain from food for 3 hours and from alcohol at least 48 hours prior to each experimental session. Breath alcohol concentration (BrAC) was measured with a breathalyzer (Draeger, Inc.) upon arrival and throughout the session when the subjects were outside the recording chamber. Since no electronic device can be used in the magnetically shielded room, BrAC was estimated with Q.E.D. Saliva Alcohol Test (OraSure Techn, Inc.) during the recording. The subjects rated their momentary moods and feelings with the Biphasic Alcohol Effects Scale, BAES [72] prior to drinking (at baseline), on the ascending BrAC and descending BrAC limbs. The mood ratings were obtained for 19 out of 20 participants. In each session, either alcohol, 0.60 g/kg for men, 0.55 g/kg for women, presented as a cocktail containing vodka (Grey Goose, Bacardi) as 20% v/v in orange juice, or placebo (the same volume of orange juice) were administered to the participants [31]. The task was administered 45±7 minutes after imbibing the beverage. In the alcohol session the average BrAC levels were 0.052±0.016 before and 0.055±0.013 after the task, suggesting that the task was administered close to the peak of the BrAC. Upon completion of each session the participants were asked to rate perceived task difficulty, type, and content of the imbibed beverage, and how intoxicated, nauseous or dizzy they felt on a 1–5 Likert scale. They were asked to estimate how many alcoholic drinks were contained in the beverage starting from 0 with 0.5 drinks increments. Transportation home was provided to all participants. High-resolution structural MRI scans were also obtained from all participants in a separate session.

### Task

A modified Stroop paradigm combined reading and color naming [31]. Subjects were asked to press a button (red, yellow, green, or blue) corresponding to the font color whenever a word was written in color (50% of the trials). In the congruent (CONG, 16.7% of all trials) condition, the meaning of the color word coincided with the font color (e.g. “red” printed in red). On incongruent (INCONG, 16.7%) trials, the meaning of the word interfered with the font color (e.g. “red” printed in blue), while on neutral (NEUT, 16.7%) trials, a common non-color word was presented in color (e.g. “paid” printed in blue). The neutral words were matched with color words in length and lexical frequency [73]. In READ condition, which comprised 50% of the trials, color words (red, green, blue, or yellow) were presented in gray and the subjects had to press a button corresponding to the meaning of the word. The READ condition was introduced with the purpose of maintaining the dominance and automaticity of the reading response [74].

Participants were instructed to respond as quickly as possible, without losing accuracy. They pressed two buttons using their index and middle fingers on each of their hands, in response to red and green (left hand) and blue and yellow colors (right hand). All words were presented for 300 ms on the black background followed by a fixation string (xxxx printed in gray) with a total trial length of 2 seconds. A total of 576 trials were presented in a random sequence with all colors equally represented in each of the four conditions. At the beginning of each session, participants practiced the task until the performance was >95%. Behavioral performance was measured by accuracy (% correct responses), reaction times (RTs), and self-corrections, defined as trials on which participants corrected themselves by pressing a correct button immediately after an erroneous response. Other behavioral measures included BAES [72] that was administered at three points during the experiment, and Likert-scale ratings of task difficulty, beverage contents, perceived level of intoxication, nausea, and dizziness that were administered upon task completion.

### Data Acquisition and Analyses

#### MRI

Structural MRI images were acquired in order to define the model for the volume conductor and the solution space for MEG source localization analysis. Images were acquired with a 3 T Siemens Trio whole-body scanner (Siemens, Erlangen). For each subject, two high-resolution 3D MP-RAGE T1-weighted sequences that optimize contrast for a range of tissue properties were obtained with the following parameters: TR = 2.53 sec, TE = 3.25 ms, flip angle = 7 degrees, FOV = 256, 128 sagittal slices, 1.33 mm thickness, in-plane resolution 1×1 mm. Structural images were used to reconstruct each person’s cortical surface [75], [76]. Inner skull surface was derived from the segmented MRI data and used for a boundary element model of the volume conductor in the forward calculations. The solution space was approximated by ∼5000 free-rotating dipoles along the gray-white matter surface in the cortex, with spacing between dipole locations ∼7 mm.

#### MEG

High-density MEG signals were recorded from 204 channels (102 pairs of planar gradiometers) with a whole-head Neuromag Vectorview system (Elekta) in a magnetically and electrically shielded room. The signals were recorded continuously with 600 Hz sampling rate and minimal filtering (0.1 to 200 Hz). The position of magnetic coils attached to the skull, the main fiduciary points such as the nose, nasion and preauricular points, as well as a large array of random points spread across the scalp were digitized with 3Space Isotrak II system for subsequent precise co-registration with structural MRI images. Trials with incorrect responses were excluded from all further analysis. In addition, the number of included trials was equated across beverage and task conditions for each subject in order to eliminate potential bias due to unequal number of trials. The MEG analysis stream primarily uses our custom Matlab functions and it relies partially on publicly available packages including FieldTrip [77], EEGLAB [78], and OpenMEEG [79]. Single trial MEG data were low-pass filtered at 100 Hz and epoched in two different ways in order to gain insight into both stimulus-related processing and response preparation and execution stages. Data were epoched from −500 ms to 1000 ms relative to each stimulus onset for the stimulus-locked analysis and from −500 to 500 ms relative to each button press for the response-locked analysis. For each epoch, the data were downsampled by a factor of 2, linear trend was removed, and mean activity across the entire epoch was subtracted from each time point. Epoched data were then passed through automatic threshold rejection to remove trials that were contaminated with eye-blinks or other artifacts. Independent component analysis [78] was used to remove heart-artifact components.

Estimated source power constrained to cortical surface was calculated based on the spectral dynamic statistical parametric mapping approach [80], by applying anatomically constrained MEG (aMEG) method based on cortically constrained minimum norm estimate [81] to the complex power spectrum. Complex power spectrum was calculated for each trial using convolution with complex Morlet wavelets [82] in 1 Hz increments from 4 to 7 Hz, with a constant time resolution of 80 ms and frequency resolution of 2 Hz. Wavelet width varied from 2 to 4 cycles in the specified frequency range to ensure constant wavelet length of 500 ms for all frequencies in the theta band. The first and last 250 ms of each epoch were discarded to remove edge artifacts potentially resulting from wavelet analysis. Theta band power was plotted for each individual epoch to further visually inspect the wavelet results and reject additional artifact contaminated trials that had not been detected via the automatic threshold rejection procedure. Complex power spectra were downsampled by a factor of 4 and entered into inverse calculations resulting in 13 ms temporal sampling rate for the spatio-temporal power estimates. To estimate the noise covariance for calculation of the inverse and to prevent biasing the inverse solution against spontaneous brain oscillations, we used empty room data that were detrended and band-pass filtered between 3 and 50 Hz. The signal-to-noise ratio (SNR) equaling 5 [80] was used for scaling of the noise covariance matrix in calculation of the inverse operator. The identity matrix was used for noise-sensitivity normalization of the source-space solution. The noise-sensitivity normalized estimates of total source power were obtained at each location on the cortical surface at each frequency. For each subject, a map of total source power was calculated by averaging across theta band frequencies (4–7 Hz) and across trials. Finally, total event-related theta power was baseline-corrected by subtracting the mean theta source power estimate in the 250 ms prestimulus period. Intersubject averages were created by morphing each subject’s reconstructed surface onto an average representation after aligning their cortical sulcal-gyral patterns [83] and averaging individual source power estimates.

Region-of-interest (ROI) analysis was conducted to further examine possible interactions of the factors of beverage, task condition, and gender on event-related changes in theta power. Unbiased ROIs were selected based on the overall group average across all subjects, task and beverage conditions and comprised dipole locations along cortical surface with most notable source power. The same set of group-based ROIs was used for all subjects in a manner blind to their individual activations by applying an automatic spherical morphing procedure [83]. The ROIs primarily encompassed the cognitive control network in the fronto-parietal regions, as shown in Figs. 2 and 3. More specifically, bilateral ROIs included dorsal ACC on the medial surface, inferior frontal junction (IFJ) [84] touching on the posterior inferior frontal sulcus and precentral sulcus, hand motor region (MOT) in the central sulcus, and intraparietal sulcus (PAR). Additionally included were the left inferior frontal gyrus (IFG) and the right superior frontal gyrus (SFG).

Repeated measures ANOVAs (SPSS for Windows, SPSS Inc) were performed with the factors of Beverage (alcohol, placebo) and Condition (CONG, INCONG, NEUT, READ) for each ROI stimulus-related theta power averaged over time points in three time windows, T1 (120–270 ms), T2 (320–470 ms), and T3 (480–670 ms), chosen to include prominent peaks in theta timecourses across ROIs, Fig. 3, Table 1. Similarly, within the response-locked analysis stream, ANOVAs were carried out on baseline-corrected response-related theta power estimated during the motor preparation time window (−200 to −50 ms), immediately preceding response execution (Fig. 4). Beverage effects on conflict-related theta power changes were assessed by comparing the difference between INCONG and CONG under alcohol and placebo for each ROI (Table 1). In addition, uncorrected baseline power estimates were submitted to the same analysis in order to examine potential effects of beverage and task condition for the −250 to 0 ms time window. Mixed design ANOVAs including Gender as a between-group factor were initially performed separately for each ROI and time window. Since no significant effects of Gender were observed in any of the analyses, reported are the results of repeated measures ANOVA with factors Condition and Beverage. Non-parametric related-samples Friedman’s ANOVA by ranks was performed when the normality assumption was violated, such as percentage of self-corrections and post-experimental Likert scale ratings. Normality was assesed using Shapiro-Wilk Test and by visual inspection using Normal Q-Q plot.

#### EEG

In order to provide a complementary measure of the effects of alcohol on event-related theta power and to relate our findings to previous EEG studies, EEG data were measured at Fz and Cz sites simultaneously with the MEG signal. An electrode placed on the tip of the nose served as the reference and the one on the right earlobe as ground. In addition, the electrooculogram (EOG) was recorded with bipolarly referred electrodes placed at the outer canthus of the left eye and just above the nasion. The electrode impedance was kept below 5 kOhms. Good quality complete EEG data sets were obtained from 17 participants and were submitted to the same signal-space analysis as described in the MEG section of the Methods. Total power was calculated at 4 to 12 Hz in 1 Hz increments using 500 ms wavelet length, 14 to 24 Hz in 2 Hz increments using 333 ms wavelet length, and 25 to 55 Hz in 5 Hz increments using 200 ms wavelet length. Grand averages of event-related power expressed as relative change to the prestimulus period were calculated (i.e. normalized power N(t,f) = (P(t,f) – B(f))/B(f), where P(t,f) is raw total power at timepoint t and frequency f and B(f) is mean power for frequency f in the prestimulus time window). Total event-related theta power was averaged for three time windows T1 (120–270 ms), T2 (320–470 ms), and T3 (480–670 ms) for each subject and entered into a repeated measures ANOVA analysis as described above.
